# Gastrointestinal Sensing of Meal-Related Signals in Humans, and Dysregulations in Eating-Related Disorders

**DOI:** 10.3390/nu11061298

**Published:** 2019-06-08

**Authors:** Maryam Hajishafiee, Vida Bitarafan, Christine Feinle-Bisset

**Affiliations:** Adelaide Medical School and National Health and Medical Research Council of Australia Centre of Research Excellence in Translating Nutritional Science to Good Health, Adelaide Health and Medical Sciences Building, Corner North Terrace and George Street, Adelaide 5005, Australia; maryam.hajishafiee@adelaide.edu.au (M.H.); vida.bitarafan@adelaide.edu.au (V.B.)

**Keywords:** gastrointestinal sensing, nutrients, gastric distension, impaired gastrointestinal function, obesity, functional dyspepsia, anorexia of ageing

## Abstract

The upper gastrointestinal (GI) tract plays a critical role in sensing the arrival of a meal, including its volume as well as nutrient and non-nutrient contents. The presence of the meal in the stomach generates a mechanical distension signal, and, as gastric emptying progresses, nutrients increasingly interact with receptors on enteroendocrine cells, triggering the release of gut hormones, with lipid and protein being particularly potent. Collectively, these signals are transmitted to the brain to regulate appetite and energy intake, or in a feedback loop relayed back to the upper GI tract to further adjust GI functions, including gastric emptying. The research in this area to date has provided important insights into how sensing of intraluminal meal-related stimuli acutely regulates appetite and energy intake in humans. However, disturbances in the detection of these stimuli have been described in a number of eating-related disorders. This paper will review the GI sensing of meal-related stimuli and the relationship with appetite and energy intake, and examine changes in GI responses to luminal stimuli in obesity, functional dyspepsia and anorexia of ageing, as examples of eating-related disorders. A much better understanding of the mechanisms underlying these dysregulations is still required to assist in the development of effective management and treatment strategies in the future.

## 1. Introduction

**Figure 1:**
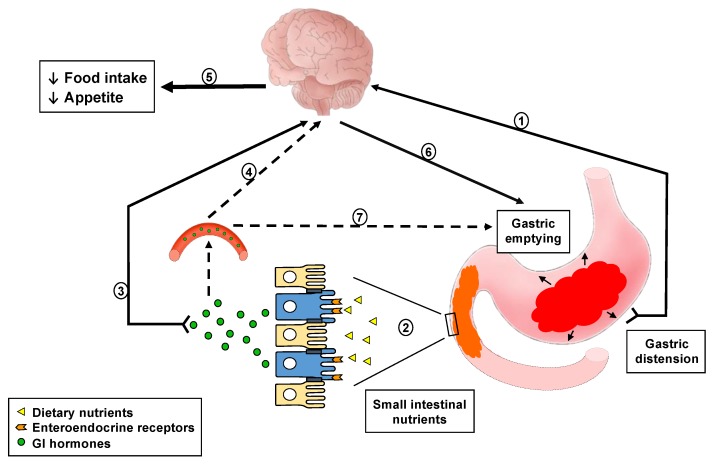
Schematic representation of the gastrointestinal (GI) sensing of meal-related stimuli, and effects on GI functions (specifically gut hormone release and slowing of gastric emptying), appetite and energy intake. Meal ingestion initially induces gastric distension, which activates mechanoreceptors on vagal afferents that terminate in the gastric wall and transmit this signal to the central nervous system (1). As chyme enters the small intestine in the process of gastric emptying, nutrients are sensed by receptors located on enteroendocrine cells, triggering GI hormone secretion (2). GI hormones convey meal-related information to the brain involving various pathways, including activation of hormone-specific receptors on vagal afferent endings (3) or following transport through the blood stream (4). Together, these inputs are conveyed to higher brain centres to modulate eating behaviour, appetite and energy intake (5), as well as feedback regulation of GI motor functions, particularly pyloric pressures, associated with the slowing of gastric emptying (6). The latter can also occur through endocrine pathways (7).

Meal ingestion is associated with well-established changes in upper gastrointestinal (GI) functions that serve to accommodate food in the stomach and break it down to particles of appropriate size for transfer into the small intestine for digestion and subsequent absorption. During these processes, the presence of food in the GI lumen generates a variety of signals arising from gastric distension, nutrient and non-nutrient compounds contained in the food, as well as gut hormones released from enteroendocrine cells in the gut wall [1,2,3,4]. Distension of the stomach, induced by the volume of food ingested, activates mechano-sensitive vagal afferent fibres with nerve endings in the submucosa and smooth muscle layers of the gastric wall [5,6,7], and gives rise to a sensation of fullness [8]. As gastric emptying progresses, the inputs from mechanical distension diminish gradually, while the small intestinal lumen is increasingly exposed to nutrients, including fats, proteins, carbohydrates and their digestion products. These are detected, or ‘sensed’, by highly specialised receptors, primarily G protein-coupled receptors, located on the luminal side of enteroendocrine cells, triggering a cascade of intracellular events to increase intracellular calcium, and culminating in the release of gut hormones from the basolateral side [2,3,9]. Gut hormones, e.g. cholecystokinin (CCK) and glucagon-like peptide-1 (GLP-1), then activate receptors located on adjacent endings of submucosal vagal afferent, as well as enteric, neurons. This information, together with the signals from gastric distension, is transmitted to the brainstem, and from there to higher centres, including the hypothalamus, to modulate eating behaviour. Within the brainstem, signals are also relayed from the nucleus of the solitary tract to the dorsal motor nucleus of the vagus, from which vagal efferents trigger feedback regulation of GI motor functions, including stimulation of pyloric pressures, leading to the slowing of gastric emptying [2,4,6,10]. Following their release from enteroendocrine cells, gut hormones are also transported in the blood stream to peripheral organs, including the stomach, where they activate specific receptors expressed on smooth muscle cells and enteric neurons, e.g., in the pylorus, to modulate gastropyloroduodenal motility associated with slowing of gastric emptying (Figure 1). Because the molecular and cellular processes involved in the sensing of these GI luminal signals cannot be investigated readily in humans, the changes in circulating concentrations of gut hormones, as well as effects on GI motor functions, including modulations in GI motility and slowing of gastric emptying, in response to these signals are frequently evaluated as ‘markers’ of GI luminal sensing in clinical studies.

While the processes outlined above underlie the regulation of normal GI function, appetite and energy intake, dysregulations can occur in a range of disorders which adversely affect eating, or lead to GI symptoms, including exaggerated postprandial fullness, nausea and bloating [11,12,13,14]. For example, in obesity, GI sensitivity to dietary fat appears to be decreased, possibly as a consequence of excess energy intake [15]. Psychiatric eating disorders, including anorexia nervosa and bulimia nervosa, have been found to be associated with an enhanced sensitivity to gastric distension and alterations in GI hormone secretion in response to nutrients [16,17]. GI disorders (including gastro-oesophageal reflux disease, and the functional GI disorders, functional dyspepsia and irritable bowel syndrome), have in common a hypersensitivity to luminal stimuli, particularly fat, triggering postprandial GI symptoms [18]. Critical illness (including sepsis, trauma, burns, head injuries or surgical emergencies in patients admitted to an intensive care unit) is also associated with hypersensitivity to small intestinal nutrients and GI motor dysfunctions, resulting in intolerance of gastric feeding [12,19]. Finally, ageing, while characterised by a reduced GI sensitivity to both fat and protein, has also been found to be associated with a range of changes in GI functions, including gastric emptying and hormone release [20], which may contribute to the characteristic loss of appetite, termed ‘anorexia of ageing’.

This review will provide a brief overview of the GI sensing of meal-related signals in humans, specifically, gastric distension and small intestinal nutrients, thus focusing on preabsorptive signals, by describing their effects on GI functions, appetite and energy intake. We will also examine dysregulations in the GI responses to these signals. While, as described above, these can occur in a wide range of disorders, a comprehensive review is beyond the scope of this paper. Thus, we will illustrate key changes, and their functional implications, using obesity, functional dyspepsia and anorexia of ageing as examples. With regards to nutrients, we will focus on fat and protein because their GI sensing is primarily altered in these disorders, as alluded to above. Thus, while carbohydrates are, of course, also sensed in the intestinal lumen [21], a discussion of their effects is beyond the scope of this paper.

## 2. GI Sensing of Intraluminal Meal-Related Stimuli

The arrival of a meal in the upper GI tract in the process of food ingestion exerts powerful signals, including gastric distension as a result of the meal volume, as well as the chemical components of the meal, particularly macronutrients, that modulate postprandial GI functions, including gastric emptying, GI motility and the release of GI hormones, associated with changes in appetite perceptions and subsequent energy intake. As outlined above, while the investigation of GI sensing of intraluminal stimuli at the receptor level is currently not feasible in human studies in-vivo, the measurement of the downstream manifestations, including changes in upper GI motility, gut hormone release, as well as appetite perceptions and energy intake, provides a relatively non-invasive means to quantify the ability to ‘sense’ these stimuli in the GI lumen in clinical research.

### 2.1. Sensing of Gastric Distension

Meal ingestion induces a gradual distension of the gastric wall, inducing fullness and acting as a first signal to control meal size [8,22,23,24]. For example, experimental distension of the proximal stomach using a bag attached to a gastric barostat gradually increases the perception of fullness, as the distension, induced either by increasing volume or pressure within the bag, increases [8,23]. Gastric distension before, or during, meal ingestion also reduces subsequent food intake [24]. Filling of the antrum also plays a role in the perception of fullness and meal termination [25]. For example, studies using scintigraphy or ultrasound to quantify intragastric volume and meal distribution showed that fullness after consumption of a 350-mL glucose drink was directly related to the volume in the distal stomach [25]. Moreover, suppression of energy intake after a mixed-nutrient drink was related inversely to antral area (a measure of antral filling) immediately before the meal [26]. The relative importance of the proximal vs distal stomach cannot be determined from these studies. However, because a meal is initially stored primarily in the proximal stomach (as a result of proximal gastric relaxation) and, in the process of gastric emptying, gradually transferred into the antrum prior to evacuation into the small intestine, it is likely that the relative importance of the two regions changes as a result of changes in the intragastric distribution of the meal as gastric emptying progresses.

### 2.2. Effects of Nutrients in the Small Intestinal Lumen

As gastric emptying progresses and the signal from gastric distension diminishes, chyme enters the small intestinal lumen in a tightly regulated fashion. This is achieved by well-characterised effects of nutrients on pressures in the antropyloroduodenal region [27], mediated by gut hormones released in response to nutrients [2,3,10]. Nutrients in the intestinal lumen, particularly lipid and protein, also modulate appetite and subsequent energy intake [28,29], and changes in both motility and gut hormones play critical roles [30,31]. 

#### 2.2.1. Small Intestinal Sensing of Lipid

The presence of lipid in the GI lumen provides a potent signal to stimulate the GI functions that are key to the regulation of appetite and energy intake [15]. For example, infusion of lipid, at loads of 1–4 kcal/min, directly into the duodenum, to exclude any sensory inputs from the oral cavity or confounding effects of variations in the rate of gastric emptying, induces well-coordinated changes in upper GI motility, including the stimulation of pyloric pressures [28], which underlie the slowing of gastric emptying [27]. Lipid also stimulates the release of GI hormones, including CCK, GLP-1 and peptide YY (PYY), while the release of ghrelin from the stomach is suppressed [10]. These effects occur in a load-dependent manner, and are associated with the suppression of energy intake [28,32]. In fact, the magnitude of the stimulation of pyloric pressures and plasma CCK, as indicators of the GI sensing of nutrients, have been identified as independent determinants of energy intake in response to intraduodenal administration of particularly fat and also protein [30,31].

The above-mentioned effects of fat on GI functions, including gastric emptying, GI motility, gut hormone release and energy intake, are abolished by co-administration of the lipase inhibitor, orlistat, establishing that the GI effects of fat are dependent on fat digestion, and lipid digestion products, namely fatty acids, are essential for intestinal lipid sensing [33,34]. Thus, the digestibility of fat affects its sensing in the GI lumen. Once fatty acids are released in the process of lipid digestion, their effects on GI functions are chain-length dependent [35]. Moreover, even within the group of fatty acids with ≥12 carbon atoms, fatty acids appear to have different potencies [36,37]. For example, only lauric acid (C12), but not oleic acid (C18:1), reduced subsequent energy intake when infused at a load of 0.4 kcal/min [36], while C18:1 was effective at the higher load of ~0.75 kcal/min [37], suggesting that the threshold loads required for luminal detection differ between fatty acids. The sensing of fatty acids in the GI lumen [38,39] is associated with the release of GI hormones, including CCK and GLP-1 [2,3], which are involved in transmitting nutrient-related information to the brain, and, in case of CCK, at least in part, via CCK-A receptor-dependent mechanisms [40,41].

#### 2.2.2. Small Intestinal Sensing of Protein

Dietary protein has been recognised to have potent effects to modulate GI functions and suppress appetite and food intake [42,43]. Amongst proteins, whey protein appears to be particularly potent [44]. For example, intraduodenal administration of whey protein, at loads of 0.5–3 kcal/min, stimulates pyloric pressures and modulates the release of gut hormones, including stimulation of CCK, GLP-1 and PYY, and suppression of ghrelin, and reduces subsequent energy intake, in a dose-dependent manner [29,45]. Moreover, the effects of whey protein, which is digested relatively rapidly, on GI hormone release, slowing of gastric emptying and suppression of energy intake are greater than those of casein, which coagulates in the stomach, suggesting a role for the digestibility of proteins in its sensing in the GI lumen [46]. Thus, as with lipids and fatty acids, amino acids may mediate, at least in part, the effects of protein on GI functions and energy intake [47,48,49]. There has, therefore, been an increased interest in evaluating the effects of specific amino acids on these outcomes [49,50]. However, the assessment of the effects of amino acids is complicated by the number of amino acids at play, their varying structures, their inter-dependence (e.g., for effective absorption) and the large range of their effects outside the GI tract. Nevertheless, a number of amino acids, when given in relatively small amounts, modulate gut functions and reduce energy intake [51,52,53,54,55]. For example, L-tryptophan, given orally, intragastrically or intraduodenally, stimulates plasma CCK and pyloric pressures, slows gastric emptying and suppresses energy intake in healthy, lean individuals [52,54,55]. In addition, the suppression of energy intake by amino acids, e.g., L-tryptophan and L-leucine, is also related to the circulating concentrations of these amino acids [51,52], in line with the recognition that the effects of amino acids on energy intake are also regulated by extraintestinal factors, which may act in the periphery and/or the brain [56]. This may explain, at least in part, why intraduodenal protein and lipid infusions have comparable effects to suppress subsequent energy intake, despite protein stimulating gut hormones and pyloric motility much less than lipid [32].

Taken together, the sensing of both lipid and protein, through their digestion products, has potent effects on GI functions, associated with a reduction in appetite and energy intake. While much work has been done in this area in humans, understanding the molecular processes involved in GI sensing still relies largely on preclinical studies, or ex-vivo investigations of clinical samples (e.g., human biopsies). Thus, technical advances are required that will enable in-vivo studies of these processes in healthy humans as well as relevant patient populations. A thorough understanding of the mechanisms underlying these processes is critical for a better understanding of the dysregulations in GI sensing underlying eating-related disorders, to develop effective management and treatment strategies.

## 3. Altered GI Sensing of Meal-Related Stimuli in Eating-Related Disorders

While, as discussed, luminal meal-related stimuli contribute to the regulation of GI functions, appetite and energy intake, disturbances in the sensing of these stimuli have been found in a number of eating-related disorders, including a reduced intestinal sensitivity to the GI effects of fat in obesity, associated with dietary overconsumption [57], an exaggerated sensitivity to both gastric distension and intestinal lipid in patients with functional dyspepsia, associated with digestive symptoms [18], and reduced GI sensory perception associated with a loss of appetite with ageing [20] (Table 1).

### 3.1. Altered GI Sensing in Obesity

People with obesity, as a group, consume larger amounts of food, and have a preference for particularly high-fat and energy-dense foods; thus, it is conceivable that their ability to sense meal-related stimuli (e.g., distension of the stomach, dietary fat) in the GI lumen is compromised.

#### 3.1.1. Sensitivity to Gastric Distension

Obese individuals have been found to have greater fasting gastric volumes [58], and in most, but not all, studies tolerate greater intragastric volumes, as measured by gradually filling a bag positioned in the stomach with air or water [59,60,61], or consume larger amounts of water or nutrient loads during drink challenges [58]. Thus, obese individuals appear to be less sensitive to gastric distension and require larger intragastric volumes to experience fullness. While data relating to gastric meal emptying in obese have been inconsistent (with studies reporting slower or faster emptying, or no differences from lean individuals [62]), possibly in part due to differences in study design and methodological approaches [15], a comprehensive recent study of 328 participants found that gastric emptying of both solid and liquid components of a mixed meal was accelerated in obese subjects [58]. Accelerated gastric emptying is associated with an enhanced exposure of the small intestine to nutrients, which has been shown to induce structural changes in the mucosa and facilitate nutrient absorption [103], therefore, differences in GI functions and energy intake in response to nutrients may be the result of reduced feedback from small intestinal nutrients, particularly fat.

#### 3.1.2. Small Intestinal Sensing of Fat

Experimental evidence that the overconsumption of energy-dense, high-fat foods is associated with a reduced GI sensitivity to fat has been derived mainly from short-term overfeeding studies, often conducted in normal-weight people [104,105,106,107]. For example, in normal-weight individuals, consumption of a high-fat diet for 2 weeks accelerated gastric emptying of a high-fat meal [104], and attenuated the pyloric motor response to an intraduodenal lipid infusion, when compared with the low-fat diet [107]. The effect on gastric emptying was fat-specific, since gastric emptying of a high-carbohydrate meal was not accelerated after the high-fat diet [104]. There is, indeed, also evidence that obese people are less sensitive to the appetite-suppressant effects of dietary fat [42,43,63,64]. For example, obese volunteers consumed a greater amount of food from a high-fat meal than healthy controls [63], and, unlike healthy controls, obese participants did not reduce subsequent energy intake after a high-fat meal [42]. Only few studies have specifically evaluated the gut hormone responses to fat ingestion in obese people, and findings are somewhat conflicting. For example, male and female obese volunteers were reported to have a greater plasma CCK response to a soup containing 30 g of margarine than healthy controls, despite comparable gastric emptying in the two groups [65], although gastric emptying of fat was not specifically quantified, and thus, may have been faster, potentially resulting in greater CCK stimulation. In contrast, we found no differences in plasma CCK between obese and lean male adults during 3 h after ingestion of a solid high-fat meal [42]. Solid meal emptying is slower than liquid emptying, possibly explaining the differences between the outcomes in the two studies; however, the latter study did not evaluate gastric emptying. Finally, we have reported reduced plasma CCK concentrations during 90-min intraduodenal administration of oleic acid in obese, compared with lean, men [66], indicating that the small intestinal response to a standardised fatty acid load is reduced in obese people, most likely due to compromised small intestinal lipid sensing. While PYY concentrations following consumption of a high-fat meal have been reported to be lower in obese than lean individuals [43], PYY and ghrelin responses to a high-fat meal have also been found to be comparable in the two groups [42]. Taken together, the limited available data suggest that obesity may be associated with a reduced ability to sense dietary fat, which may compromise the initiation of appropriate feedback mechanisms, including gut hormone responses. Fat-induced gut hormone secretion may be reduced, or, in the case of normal secretion, the sensitivity to hormones may be compromised, and these changes may contribute to altered energy intake regulation.

There is also limited evidence that dietary restriction can, at least in part, improve intestinal responses to intraluminal fat in obesity [108,109], further supporting a contributory role of diet. For example, in obese volunteers, dietary restriction for 4 days (~1000 kcal/day) significantly enhanced plasma PYY, ghrelin suppression and pyloric contractions in response to intraduodenal lipid, associated with suppression of energy intake [108]. Moreover, 30% dietary restriction for 12 weeks was associated with greater intraduodenal lipid-induced stimulation of PYY and basal pyloric pressures, and reduced energy intake [109].

#### 3.1.3. Small Intestinal Sensing of Protein

In contrast to lipids, obese people appear to remain sensitive to the GI and appetite-suppressant effects of protein, also evidenced by the potent effects of high-protein diets to achieve weight loss [110,111]. For example, energy intake 3 h after a high-protein meal was lower than after a high-carbohydrate control meal, while (as discussed above), a high-fat meal did not reduce subsequent intake [42]. Similarly, a high-protein meal reduced hunger perceptions for 3 h post-meal substantially more than a high-fat meal, and the response to protein did not differ from those in lean participants [43]. These effects of protein may be mediated, at least in part, by gut hormones; however, current evidence is limited and inconsistent. For example, the potent suppression of hunger by the high-protein meal was accompanied by marked stimulation of plasma PYY, although absolute concentrations were lower in the obese than in the lean group, while no differences in plasma GLP-1 or ghrelin concentrations were observed between groups [43]. In contrast, in the other study [42], the high-protein meal led to sustained CCK stimulation and ghrelin suppression in both lean and obese, while the PYY response did not differ between the high-protein and high-fat meal in lean or obese. Nevertheless, that these responses are, at least in part, mediated from the small intestine, is supported by a recent study [112] in which the antropyloroduodenal pressure, plasma CCK and GLP-1 responses to intraduodenal whey protein, at the load of 3-kcal/min, did not differ between lean and obese subjects, although energy intake was non-significantly higher in the obese.

The role of specific amino acids in the responses to protein is currently unclear, with limited information on the comparative effects of amino acids on GI functions and energy intake in health and obesity. Intraduodenal infusion of tryptophan had comparable effects on pyloroduodenal motility in lean and overweight participants [113], and intragastrically administered tryptophan slowed gastric emptying and reduced energy intake after a mixed-nutrient drink in ~50% of lean and obese individuals [114]. In contrast, obese individuals have been reported to be less able to detect glutamate orally [115], suggesting that obese individuals may be less sensitive to palatable umami taste, which may contribute to higher food intakes.

Taken together, obese people appear to be less sensitive to gastric distension, and the GI and appetite-suppressant effects of fat, possibly as a result of overconsumption of high-fat, energy-dense diets, while the responses to protein remain relatively intact. Further research is needed to elucidate the mechanisms that underlie these changes, and the differential responses to protein and fat, as well as the responses to dietary restriction, at the level of the receptors, and along the pathways that transmit the information to the brain, to develop novel, and effective, strategies to better manage or treat, and ideally prevent, obesity.

### 3.2. Altered GI Sensing in Functional Dyspepsia

Functional dyspepsia (FD) is a multi-factorial disorder characterised by symptoms, including nausea, fullness, discomfort, bloating and vomiting, originating in the upper GI region, often triggered in close temporal association with meal ingestion, with patients unable to complete normal-sized meals [11,116]. This originally led to the assumption that FD was due to abnormalities in GI motor activity and gastric emptying; however, correlations between symptoms and changes in these functions are not strong. A number of contributing factors and mechanisms have been identified in FD, including gastroduodenal inflammation and changes in the epithelial barrier, GI infections, gut microbiota, genetic contributions, cognitive and psychological factors [73,117], and a key feature is an increased GI sensitivity to meal-related stimuli, including gastric distension (potentially exacerbated by delayed gastric emptying, impaired proximal stomach accommodation, abnormal intragastric meal distribution and disordered antroduodenal motor function) and/or small intestinal nutrients [73].

#### 3.2.1. Sensitivity to Gastric Distension

The frequent occurrence of FD symptoms in close temporal association with meal ingestion [74,118] suggested an enhanced sensitivity to distension of the stomach by the meal volume. Indeed, studies evaluating the gastric sensory response to gastric distension have revealed that 30–48% of patients exhibit a hypersensitivity to mechanical distension of the stomach [75,76]. Thus, when either the proximal [75,76] or distal [77] stomach was distended with an air-filled bag, FD patients reported both perception and discomfort at lower distension volumes or pressures than healthy controls. This hypersensitivity to gastric distension is also likely to underlie the inability of FD patients to complete normal-sized meals. 

#### 3.2.2. Alterations in the Small Intestinal Sensing of Nutrients

The frequent complaints by FD patients that certain foods or meals induce, or exacerbate, their symptoms suggest that FD might also be associated with a hypersensitivity to specific nutrients or other food components. Since rich and fatty foods appear to be particularly potent in triggering dyspeptic symptoms [11,73], a body of research has investigated a specific hypersensitivity to fat. However, a range of other foods, or food groups, are also frequently reported by patients to lead to symptoms [73,119,120,121,122], including milk and dairy products, meat, carbohydrate- or wheat-containing foods or drinks, certain vegetables (possible particularly those vegetables containing fermentable oligo-, di- and mono-saccharides and polyols, or ‘FODMAPs’ [123]), sour, acid-secreting or irritant foods, including citrus fruit, spices, coffee and alcohol [120,121,124]. Thus, in addition to an enhanced fat sensitivity, hypersensitivities to other nutrients or food components may also exist.

Hypersensitivity to lipid: Approximately 60–70% of FD patients display a hypersensitivity to fat [72,78,79,80]. For example, dyspeptic symptoms, including epigastric pain, bloating and nausea, were substantially greater in response to a high-fat soup than a bland soup [80]. Similarly, a palatable high-fat yogurt was associated with significantly greater fullness, nausea and bloating than an equivolaemic fat-free yogurt [78,79]. The importance of a contribution from the small intestine is highlighted by the fact that intraduodenal infusion of a long-chain triglyceride emulsion induced typical symptoms, and exacerbated the sensitivity to gastric distension, in patients, but not healthy controls [72]. This hypersensitivity appears to be fat-specific, since infusion of glucose did not induce symptoms [81]. Moreover, administration of the CCK-A receptor antagonist, dexloxiglumide, reduced nausea, bloating and fullness, induced by duodenal lipid infusion, in patients [82], providing evidence that CCK mediates, at least in part, the effects of fat on symptoms in FD. Whether FD is associated with a hypersensitivity to [82,83], or altered secretion of [79], CCK, or both, and the involvement of other gut hormones, remains unclear and warrants investigation.

Responses to other nutrients: Since protein, similarly to fat, potently affects upper GI functions and energy intake in healthy people, it is conceivable that protein consumption could also generate FD symptoms; however, this has not been investigated. One study quantifying eating habits and the temporal relationship with dyspeptic symptoms in FD over one week indicated that although there was no difference in dietary protein consumption between FD and healthy individuals, postprandial fullness was related to protein in the patients [74]. Moreover, some patients report dyspeptic symptoms after consumption of wheat-containing foods [119,120,125], which may be related to gluten [126,127], and a gluten-free diet has been found to reduce dyspeptic symptoms [127]. However, it is not clear whether such findings relate specifically to an intolerance of gluten or, more broadly, to other protein sources.

Findings relating to effects of different sources of carbohydrate on FD symptoms are limited. One study reported inverse relationships between overall symptoms and fullness with carbohydrate intake [74], suggesting that carbohydrates overall play a favourable role. The role of dietary fibre in FD is still uncertain [120,128]. No studies have evaluated the role of FODMAPs, or their elimination from the diet, in FD. Symptoms reported in response to milk ingestion may be due to lactose intolerance, or relate to the fat or protein content of milk, but this requires further study [120].

Taken together, FD is associated with hypersensitivities to both gastric distension and small intestinal nutrients, particularly fat; thus, these disturbances may, at least in part, address the patients’ frequent complaints of an inability to complete normal-sized meals and intolerance of fatty foods. Much more research is required to clarify the contributions of a large range of other foods, and food components, including protein, to FD symptoms, and mechanisms involved, ideally in large studies to allow sub-grouping of patients. Such approaches are vital, as they may eventually enable the development of specific dietary interventions for translation into effective therapeutic strategies.

### 3.3. Altered GI Sensing in Anorexia of Ageing

Ageing, even in healthy people, is often associated with a loss of appetite, termed “anorexia of ageing”. Older people consume smaller meals and fewer snacks, and eat more slowly, compared with young adults [129], resulting in a decline in energy intake and weight loss. Chronic weight-loss represents a major risk to the health and well-being of older people, hence, nutritional strategies, which include particularly the use of protein supplements, have been developed to address this problem [130]. While the causes of appetite loss with ageing are not completely understood, ageing is associated with a gradual decline in metabolically active tissue, specifically muscle mass [130]; thus, a reduction in basal metabolic rate, associated with reduced energy requirements, may lead to reduced appetite. However, there is evidence of altered GI sensory and motor functions, including slower gastric emptying [20], which would favour a reduction in energy intake (but also delays initiation of signals by nutrients in the small intestine), as well as, on the other hand, a reduced sensitivity to the energy intake-suppressant effects of nutrients [87,88,131], and changes in the secretion of, and/or sensitivity to, gut hormones [89]. An improved understanding of these, apparently discrepant, changes in gastric function vs appetite signals arising from the small intestine in response to meal consumption is likely to assist in the development of improved management strategies to ensure that older people receive adequate nutrition.

#### 3.3.1. Sensitivity to Gastric Distension

Older people frequently report reduced appetite before and during meal ingestion. For example, healthy older people were less hungry before and following the ingestion of a mixed-nutrient yogurt-based drink, and reported greater fullness after the drink than young controls [26]. Early studies evaluated gastric emptying of meals and most [90,91,92,93], but not all [132], found that gastric emptying of both solid and liquid meal phases was slower in older than young people, although observed differences were often modest [91,93]. Increased gastric meal retention may enhance gastric distension in either proximal or distal stomach contributing to fullness, as described in healthy people [23,25]. While proximal and/or distal gastric retention has been found to be greater [26,93], antral filling has also been reported to be less [90], in older people. In response to isovolumetric or isobaric proximal gastric distension, older people reported less fullness or bloating, and greater hunger, than young controls, at a given volume or pressure level, in the absence of any changes in gastric compliance [94], suggesting that healthy ageing is associated with a reduced perception of gastric distension. Reasons for the apparent discrepancies between responses to a meal, as opposed to experimental gastric distension, are currently unclear.

#### 3.3.2. Alterations in the Small Intestinal Sensing of Nutrients

The effects of intestinal nutrient exposure, with a focus on protein, on GI functions and appetite in older people have been evaluated in a limited number of studies, and findings suggest that ageing is associated with a reduced responsiveness to the appetite-suppressant effects of nutrients [87,95,133,134]. This may be due, at least in part, to a reduced digestive capacity with ageing, since reductions in the secretions of gastric acid, pancreatic lipase and other enzymes, as well as bile salts have been reported [135]; however, whether these changes have any detrimental effects on the digestion of protein and fat, and whether, or how, that may alter the GI sensing of these nutrients, requires investigation.

Response to protein: In contrast to the use of high-protein diets to achieve weight loss in obesity [110,111], in older people protein supplements are recommended to prevent weight loss and maintain functionality [130]. Given the potent GI and appetite-suppressant effects of protein in young people [42,43,136], it is important to increase our knowledge of the alterations in the GI effects of protein in older people, and underlying mechanisms. The available literature indicates that ageing is associated with a reduced sensitivity to the satiating effect of protein [87,88,95]. For example, despite a reduced desire to eat, as well as reduced fullness, in response to a protein drink containing either 30 g or 70 g whey protein, the suppression of energy intake relative to control from a meal consumed 180 min later was less in older people, associated with a greater cumulative intake [87]. Interestingly, despite slower gastric emptying of the drinks in older people, energy intake from the meal was only related to gastric emptying in the younger people [87]. Similarly, a 60-min intraduodenal infusion of whey protein suppressed appetite and energy intake less in healthy older than in young adults, associated with greater overall energy intake in older people [95]. Moreover, ingestion of either a whey protein drink (70 g protein; 280 kcal), or a mixed-nutrient drink (70 g protein, 28 g carbohydrate, 12.4 g fat; 504 kcal) did not suppress energy intake differentially, so that total energy intake was increased, and most by the higher-energy mixed-nutrient drink [88].

Response to lipid: Only few studies have evaluated the effects of fat on appetite perception in ageing. For example, administration of a fat emulsion (30 mL, 120 kcal) 3 times/day for six weeks significantly increased daily energy intake by ~240 kcal [137], and either a high-fat or high-carbohydrate mixed-nutrient drink (250 mL, ~250 kcal) consumed after breakfast increased intake over the following 24 h by ~200 kcal [131], with no differences between fat and carbohydrate-rich drinks. One earlier study evaluated the effects of ageing on the pyloric motor, appetite and energy intake responses to duodenal lipid and glucose infusion [96]. Lipid stimulated pyloric pressures more in older people (which is likely to underlie the slower gastric emptying described above), and while baseline hunger was less in older people, and, unlike in young controls, not suppressed by either nutrient, subsequent energy intake did not differ between the two groups.

Collectively, these studies suggest that older people are less sensitive to the appetite-suppressant effects, but more sensitive to the inhibitory effects on the stomach, particularly gastric emptying, of small intestinal nutrients. It is possible that these changes are due to alterations in the release of, or sensitivity to, GI hormones.

Gut hormone responses: Available studies consistently report increased fasting plasma CCK concentrations [89,90,97,98], as well as an exaggerated rise in response to oral or intraduodenal nutrients [88,90,97,99], in older people. Furthermore, an inverse relationship between hunger and plasma CCK has been found in young, but not older people [97], and exogenous administration of CCK-8 suppressed food intake from a meal after the infusion twice as much in older, than young people [89], suggesting that older people remain responsive to CCK and their sensitivity to the appetite-suppressant effect of CCK may be enhanced. It is not known whether the stomach remains sensitive to CCK with ageing; a reduced gallbladder contraction and emptying has been reported previously [90]. Studies evaluating the secretion of GLP-1 and PYY have yielded more inconsistent findings, with some studies reporting no differences in GLP-1 or PYY between older and young people [97,98,99], or lower [45] or greater [88,90] levels in older people. It is conceivable, but has not been investigated, that older people may be more sensitive to the effects of GLP-1 and/or PYY, resulting in an exaggerated ileal brake effect from the distal small intestine [138]. The effects of ageing on ghrelin secretion are also unclear, with studies reporting greater [100] or lower [101] fasting acyl-ghrelin, greater fasting total ghrelin [98], no difference in fasting or postprandial ghrelin [102], or lower postprandial total ghrelin [88], in older and young people.

Taken together, current evidence suggests that even healthy ageing is associated with marked changes in upper GI functions, including delayed gastric emptying and a heightened sensitivity to particularly CCK, both of which would favour suppression of appetite and energy intake. However, and in apparent contrast, the appetite-suppressant effects of nutrients are reduced in older people. While the latter lends support to the utility of dietary supplements to improve energy intake in older people, the discrepancy between these findings and the consistently reported lack of appetite frequently leading to undernutrition in older people requires much further research to identify mechanisms, and other factors, that may help to explain the apparent divergence in current knowledge, with the aim to develop improved management strategies.

## 4. Summary and Future Directions

This article has reviewed the sensing of meal-related signals, including both mechanical and nutrient stimuli, in the upper GI tract, and their effects to modulate GI functions, appetite and energy intake, in humans. The appropriate sensing of these stimuli is altered in a number of eating-related disorders, including obesity, functional dyspepsia and anorexia of ageing, associated with compromised, or exaggerated, responses to meals. In obesity, there is evidence of an enhanced gastric capacity and reduced luminal sensing of gastric distension and duodenal lipid, associated with reduced inhibition of subsequent energy intake. Functional dyspepsia, on the other hand, is associated with hypersensitivity to both gastric distension and small intestinal lipid, amongst other food components, which, at least in part, underlies the induction of meal-related symptoms, particularly in response to fatty foods. Anorexia of ageing is characterised by reduced hunger perception and food intake, in part due to delayed gastric meal emptying and an enhanced secretion of, and/or sensitivity to, gut hormones, particularly CCK. In contrast, the satiating effects of nutrients are reduced, associated, in an apparent discrepancy to the free-living situation, with an increase in overall energy intake in the laboratory setting. These examples demonstrate the existence of a variety of sensory dysfunctions across eating-related disorders that may, at least in part, underlie the changes in food intake, or symptoms experienced, in these conditions. Much more research is required on the cause-effect relationships to better understand whether the sensory changes are causal, or occur as a result of particular dietary behaviours. For example, is over-eating in obesity the result of an inherently reduced GI sensitivity to meal-related stimuli, or does gradual over-eating lead to a desensitisation of the sensory systems with subsequent reductions in the ability to adequately sense these stimuli? The temporal relationship between the decline in GI sensitivity to meal-related stimuli and reduced basal metabolic rate with ageing also warrants investigation. In functional dyspepsia, studies in large cohorts are required to enable much more detailed investigations of the varied responses to different food groups, and how these may relate to specific changes in small intestinal nutrient sensing. Further technological advances will be required to investigate the alterations that occur in these disorders at the molecular and cellular levels in vivo, and to clarify the locations of the dysregulations along both directions of the gut-brain axis. While our knowledge in this field has advanced rapidly over the last decade, much more work is still required in order to develop novel and effective approaches for the management, treatment and/or prevention of these dysregulations in GI luminal sensing.

## Figures and Tables

**Table 1:** Changes in upper GI luminal sensing that have been described in eating-related disorders.

	GI Sensory Disturbances	References
Obesity	↓ Sensitivity to gastric distension ↑ Gastric capacity ↓ Sensitivity to small intestinal lipid ?↓ Sensitivity to CCK, PYY, ghrelin ↑ ↓ ↔ CCK secretion ↑ ↓ ↔ Gastric emptying	[42,43,57,58,59,60,61,62,63,64,65,66]
Psychiatric eating disorders		
Anorexia nervosa	↑ Sensitivity to gastric distension ↑ Sensitivity to small intestinal nutrients ↑ ↔ CCK secretion ?↑ Sensitivity to CCK ↑ Ghrelin, PYY secretion ↓ Gastric emptying ↓ Proximal gastric accommodation ↑ Antral filling	[13,16,17,67]
Bulimia nervosa	↑ Sensitivity to gastric distension ↑ Gastric capacity ↓ CCK secretion ↓ ↔ Gastric emptying ↓ Proximal gastric accommodation	[16,17,68]
GI disorders		
Gastroesophagealreflux disease	↑ Sensitivity to gastric distension ↑ Sensitivity to small intestinal lipid ↓ Gastric emptying ↓ Gastric accommodation	[18,69,70,71]
Functional dyspepsia	↑ Sensitivity to gastric distension ↑ Sensitivity to small intestinal lipid ?↑ Sensitivity to CCK ↓ Gastric emptying ↓ Gastric accommodation ↑ Antral distension	[11,18,72,73,74,75,76,77,78,79,80,81,82,83]
Irritable bowelsyndrome	↑ Sensitivity to gastric distension ↑ Sensitivity to small intestinal lipid ↓ Gastric emptying ↑ ↓ Gut motility	[18,84,85]
Critical illness	↓ Sensitivity to gastric distension ↑ Sensitivity to small intestinal nutrients ↑ CCK, PYY secretion ↓ Ghrelin, motilin secretion ↓ Gastric emptying	[12,19,86]
Anorexia of ageing	↓ Sensitivity to gastric distension ↓ Sensitivity to small intestinal nutrients ↑ CCK secretion ↑ ↓ ↔ PYY, GLP-1 and ghrelin secretion ?↓ Sensitivity to CCK, PYY and GLP-1 ↓ ↔ Gastric emptying ↑ Proximal gastric retention	[26,87,88,89,90,91,92,93,94,95,96,97,98,99,100,101,102]

↑, increase; ↓, decrease; ↔, unchanged; ?, uncertain; GI, gastrointestinal; CCK, cholecystokinin; PYY, peptide YY; GLP-1, glucagon-like peptide-1.

## References

[1] Vella A., Camilleri M. (2017). The gastrointestinal tract as an integrator of mechanical and hormonal response to nutrient ingestion. Diabetes.

[2] Latorre R., Sternini C., De Giorgio R., Greenwood-Van Meerveld B. (2016). Enteroendocrine cells: A review of their role in brain-gut communication. Neurogastroenterol. Motil..

[3] Depoortere I. (2014). Taste receptors of the gut: Emerging roles in health and disease. Gut.

[4] De Lartigue G., Diepenbroek C. (2016). Novel developments in vagal afferent nutrient sensing and its role in energy homeostasis. Curr. Opin. Pharmacol..

[5] Grundy D. (2006). Signalling the state of the digestive tract. Auton. Neurosci..

[6] Furness J.B., Rivera L.R., Cho H.J., Bravo D.M., Callaghan B. (2013). The gut as a sensory organ. Nat. Rev. Gastroenterol. Hepatol..

[7] Brookes S.J., Spencer N.J., Costa M., Zagorodnyuk V.P. (2013). Extrinsic primary afferent signalling in the gut. Nat. Rev. Gastroenterol. Hepatol..

[8] Feinle C., Grundy D., Read N.W. (1997). Effects of duodenal nutrients on sensory and motor responses of the human stomach to distension. Am. J. Physiol..

[9] Ezcurra M., Reimann F., Gribble F.M., Emery E. (2013). Molecular mechanisms of incretin hormone secretion. Curr. Opin. Pharmacol..

[10] Steinert R.E., Feinle-Bisset C., Asarian L., Horowitz M., Beglinger C., Geary N. (2017). Ghrelin, CCK, GLP-1, and PYY(3-36): Secretory controls and physiological roles in eating and glycemia in health, obesity, and after RYGB. Physiol. Rev..

[11] Enck P., Azpiroz F., Boeckxstaens G., Elsenbruch S., Feinle-Bisset C., Holtmann G., Lackner J.M., Ronkainen J., Schemann M., Stengel A. (2017). Functional dyspepsia. Nat. Rev. Dis. Primers.

[12] Deane A., Chapman M.J., Fraser R.J., Horowitz M. (2010). Bench-to-bedside review: The gut as an endocrine organ in the critically ill. Crit. Care.

[13] Monteleone P., Castaldo E., Maj M. (2008). Neuroendocrine dysregulation of food intake in eating disorders. Regul. Pept..

[14] Suzuki K., Jayasena C.N., Bloom S.R. (2012). Obesity and appetite control. Exp. Diabetes. Res..

[15] Little T.J., Feinle-Bisset C. (2011). Effects of dietary fat on appetite and energy intake in health and obesity--oral and gastrointestinal sensory contributions. Physiol. Behav..

[16] Hetterich L., Mack I., Giel K.E., Zipfel S., Stengel A. (2018). An update on gastrointestinal disturbances in eating disorders. Mol. Cell. Endocrinol..

[17] Tong J., D’Alessio D. (2011). Eating disorders and gastrointestinal peptides. Curr. Opin. Endocrinol. Diabetes Obes..

[18] Feinle-Bisset C., Azpiroz F. (2013). Dietary lipids and functional gastrointestinal disorders. Am. J. Gastroenterol..

[19] Chapman M.J., Deane A.M. (2015). Gastrointestinal dysfunction relating to the provision of nutrition in the critically ill. Curr. Opin. Clin. Nutr. Metab. Care.

[20] Parker B.A., Chapman I.M. (2004). Food intake and ageing—The role of the gut. Mech. Ageing. Dev..

[21] Kreuch D., Keating D.J., Wu T., Horowitz M., Rayner C.K., Young R.L. (2018). Gut mechanisms linking intestinal sweet sensing to glycemic control. Front. Endocrinol..

[22] Wang G.J., Tomasi D., Backus W., Wang R., Telang F., Geliebter A., Korner J., Bauman A., Fowler J.S., Thanos P.K. (2008). Gastric distention activates satiety circuitry in the human brain. Neuroimage.

[23] Distrutti E., Azpiroz F., Soldevilla A., Malagelada J.R. (1999). Gastric wall tension determines perception of gastric distention. Gastroenterology.

[24] Kissileff H.R., Carretta J.C., Geliebter A., Pi-Sunyer F.X. (2003). Cholecystokinin and stomach distension combine to reduce food intake in humans. Am. J. Physiol. Regul. Integr. Comp. Physiol..

[25] Jones K.L., Doran S.M., Hveem K., Bartholomeusz F.D., Morley J.E., Sun W.M., Chatterton B.E., Horowitz M. (1997). Relation between postprandial satiation and antral area in normal subjects. Am. J. Clin. Nutr..

[26] Sturm K., Parker B., Wishart J., Feinle-Bisset C., Jones K.L., Chapman I., Horowitz M. (2004). Energy intake and appetite are related to antral area in healthy young and older subjects. Am. J. Clin. Nutr..

[27] Heddle R., Collins P.J., Dent J., Horowitz M., Read N.W., Chatterton B., Houghton L.A. (1989). Motor mechanisms associated with slowing of the gastric emptying of a solid meal by an intraduodenal lipid infusion. J. Gastroenterol. Hepatol..

[28] Pilichiewicz A.N., Papadopoulos P., Brennan I.M., Little T.J., Meyer J.H., Wishart J.M., Horowitz M., Feinle-Bisset C. (2007). Load-dependent effects of duodenal lipid on antropyloroduodenal motility, plasma CCK and PYY, and energy intake in healthy men. Am. J. Physiol. Regul. Integr. Comp. Physiol..

[29] Ryan A.T., Feinle-Bisset C., Kallas A., Wishart J.M., Clifton P.M., Horowitz M., Luscombe-Marsh N.D. (2012). Intraduodenal protein modulates antropyloroduodenal motility, hormone release, glycemia, appetite, and energy intake in lean men. Am. J. Clin. Nutr..

[30] Schober G., Lange K., Steinert R.E., Hutchison A.T., Luscombe-Marsh N.D., Landrock M.F., Horowitz M., Seimon R.V., Feinle-Bisset C. (2016). Contributions of upper gut hormones and motility to the energy intake-suppressant effects of intraduodenal nutrients in healthy, lean men—A pooled-data analysis. Physiol. Rep..

[31] Seimon R.V., Lange K., Little T.J., Brennan I.M., Pilichiewicz A.N., Feltrin K.L., Smeets A.J., Horowitz M., Feinle-Bisset C. (2010). Pooled-data analysis identifies pyloric pressures and plasma cholecystokinin concentrations as major determinants of acute energy intake in healthy, lean men. Am. J. Clin. Nutr..

[32] Ryan A.T., Luscombe-Marsh N.D., Saies A.A., Little T.J., Standfield S., Horowitz M., Feinle-Bisset C. (2013). Effects of intraduodenal lipid and protein on gut motility and hormone release, glycemia, appetite, and energy intake in lean men. Am. J. Clin. Nutr..

[33] Feinle C., O’Donovan D., Doran S., Andrews J.M., Wishart J., Chapman I., Horowitz M. (2003). Effects of fat digestion on appetite, APD motility, and gut hormones in response to duodenal fat infusion in humans. Am. J. Physiol. Gastrointest. Liver Physiol..

[34] Beglinger S., Drewe J., Schirra J., Goke B., D’Amato M., Beglinger C. (2010). Role of fat hydrolysis in regulating glucagon-like peptide-1 secretion. J. Clin. Endocrinol. Metab..

[35] Hunt J.N., Knox M.T. (1968). A relation between the chain length of fatty acids and the slowing of gastric emptying. J. Physiol..

[36] Feltrin K.L., Little T.J., Meyer J.H., Horowitz M., Rades T., Wishart J., Feinle-Bisset C. (2008). Comparative effects of intraduodenal infusions of lauric and oleic acids on antropyloroduodenal motility, plasma cholecystokinin and peptide YY, appetite, and energy intake in healthy men. Am. J. Clin. Nutr..

[37] Matzinger D., Degen L., Drewe J., Meuli J., Duebendorfer R., Ruckstuhl N., D’Amato M., Rovati L., Beglinger C. (2000). The role of long chain fatty acids in regulating food intake and cholecystokinin release in humans. Gut.

[38] Miyauchi S., Hirasawa A., Ichimura A., Hara T., Tsujimoto G. (2010). New frontiers in gut nutrient sensor research: Free fatty acid sensing in the gastrointestinal tract. J. Pharmacol. Sci..

[39] Cvijanovic N., Isaacs N.J., Rayner C.K., Feinle-Bisset C., Young R.L., Little T.J. (2017). Lipid stimulation of fatty acid sensors in the human duodenum: Relationship with gastrointestinal hormones, BMI and diet. Int. J. Obes..

[40] Feinle C., D’Amato M., Read N.W. (1996). Cholecystokinin-A receptors modulate gastric sensory and motor responses to gastric distension and duodenal lipid. Gastroenterology.

[41] Lassman D.J., McKie S., Gregory L.J., Lal S., D’Amato M., Steele I., Varro A., Dockray G.J., Williams S.C., Thompson D.G. (2010). Defining the role of cholecystokinin in the lipid-induced human brain activation matrix. Gastroenterology.

[42] Brennan I.M., Luscombe-Marsh N.D., Seimon R.V., Otto B., Horowitz M., Wishart J.M., Feinle-Bisset C. (2012). Effects of fat, protein, and carbohydrate and protein load on appetite, plasma cholecystokinin, peptide YY, and ghrelin, and energy intake in lean and obese men. Am. J. Physiol. Gastrointest. Liver Physiol..

[43] Batterham R.L., Heffron H., Kapoor S., Chivers J.E., Chandarana K., Herzog H., Le Roux C.W., Thomas E.L., Bell J.D., Withers D.J. (2006). Critical role for peptide YY in protein-mediated satiation and body-weight regulation. Cell. Metab..

[44] Anderson G.H., Tecimer S.N., Shah D., Zafar T.A. (2004). Protein source, quantity, and time of consumption determine the effect of proteins on short-term food intake in young men. J. Nutr..

[45] Giezenaar C., Luscombe-Marsh N.D., Hutchison A.T., Standfield S., Feinle-Bisset C., Horowitz M., Chapman I., Soenen S. (2018). Dose-dependent effects of randomized intraduodenal whey-protein loads on glucose, gut hormone, and amino acid concentrations in healthy older and younger men. Nutrients.

[46] Hall W.L., Millward D.J., Long S.J., Morgan L.M. (2003). Casein and whey exert different effects on plasma amino acid profiles, gastrointestinal hormone secretion and appetite. Br. J. Nutr..

[47] Thimister P.W., Hopman W.P., Sloots C.E., Rosenbusch G., Willems H.L., Trijbels F.J., Jansen J.B. (1996). Role of intraduodenal proteases in plasma cholecystokinin and pancreaticobiliary responses to protein and amino acids. Gastroenterology.

[48] Luscombe-Marsh N.D., Hutchison A.T., Soenen S., Steinert R.E., Clifton P.M., Horowitz M., Feinle-Bisset C. (2016). Plasma free amino acid responses to intraduodenal whey protein, and relationships with insulin, glucagon-like peptide-1 and energy intake in lean healthy men. Nutrients.

[49] Mellinkoff S.M., Frankland M., Boyle D., Greipel M. (1997). Relationship between serum amino acid concentration and fluctuations in appetite. 1956. Obes. Res..

[50] Steinert R.E., Ullrich S.S., Geary N., Asarian L., Bueter M., Horowitz M., Feinle-Bisset C. (2017). Comparative effects of intraduodenal amino acid infusions on food intake and gut hormone release in healthy males. Physiol. Rep..

[51] Steinert R.E., Landrock M.F., Ullrich S.S., Standfield S., Otto B., Horowitz M., Feinle-Bisset C. (2015). Effects of intraduodenal infusion of the branched-chain amino acid leucine on ad libitum eating, gut motor and hormone functions, and glycemia in healthy men. Am. J. Clin. Nutr..

[52] Steinert R.E., Luscombe-Marsh N.D., Little T.J., Standfield S., Otto B., Horowitz M., Feinle-Bisset C. (2014). Effects of intraduodenal infusion of L-tryptophan on ad libitum eating, antropyloroduodenal motility, glycemia, insulinemia, and gut peptide secretion in healthy men. J. Clin. Endocrinol. Metab..

[53] Ballinger A.B., Clark M.L. (1994). L-phenylalanine releases cholecystokinin (CCK) and is associated with reduced food intake in humans: Evidence for a physiological role of CCK in control of eating. Metabolism.

[54] Meyer-Gerspach A.C., Hafliger S., Meili J., Doody A., Rehfeld J.F., Drewe J., Beglinger C., Wolnerhanssen B. (2016). Effect of L-tryptophan and L-leucine on gut hormone secretion, appetite feelings and gastric emptying rates in lean and non-diabetic obese participants: A randomized, double-blind, parallel-group trial. PLoS ONE.

[55] Carney B.I., Jones K.L., Horowitz M., Sun W.M., Hebbard G., Edelbroek M.A. (1994). Stereospecific effects of tryptophan on gastric emptying and hunger in humans. J. Gastroenterol. Hepatol..

[56] Potier M., Darcel N., Tome D. (2009). Protein, amino acids and the control of food intake. Curr. Opin. Clin. Nutr. Metab. Care.

[57] Stewart J.E., Feinle-Bisset C., Keast R.S. (2011). Fatty acid detection during food consumption and digestion: Associations with ingestive behavior and obesity. Prog. Lipid. Res..

[58] Acosta A., Camilleri M., Shin A., Vazquez-Roque M.I., Iturrino J., Burton D., O’Neill J., Eckert D., Zinsmeister A.R. (2015). Quantitative gastrointestinal and psychological traits associated with obesity and response to weight-loss therapy. Gastroenterology.

[59] Geliebter A., Schachter S., Lohmann-Walter C., Feldman H., Hashim S.A. (1996). Reduced stomach capacity in obese subjects after dieting. Am. J. Clin. Nutr..

[60] Granstrom L., Backman L. (1985). Stomach distension in extremely obese and in normal subjects. Acta. Chir. Scand..

[61] Geliebter A. (1988). Gastric distension and gastric capacity in relation to food intake in humans. Physiol. Behav..

[62] Park M.I., Camilleri M. (2005). Gastric motor and sensory functions in obesity. Obes. Res..

[63] Speechly D.P., Buffenstein R. (2000). Appetite dysfunction in obese males: Evidence for role of hyperinsulinaemia in passive overconsumption with a high fat diet. Eur. J. Clin. Nutr..

[64] Rolls B.J., Kim-Harris S., Fischman M.W., Foltin R.W., Moran T.H., Stoner S.A. (1994). Satiety after preloads with different amounts of fat and carbohydrate: Implications for obesity. Am. J. Clin. Nutr..

[65] French S.J., Murray B., Rumsey R.D., Sepple C.P., Read N.W. (1993). Preliminary studies on the gastrointestinal responses to fatty meals in obese people. Int. J. Obes. Relat. Metab. Disord..

[66] Stewart J.E., Seimon R.V., Otto B., Keast R.S., Clifton P.M., Feinle-Bisset C. (2011). Marked differences in gustatory and gastrointestinal sensitivity to oleic acid between lean and obese men. Am. J. Clin. Nutr..

[67] Heruc G.A., Little T.J., Kohn M., Madden S., Clarke S., Horowitz M., Feinle-Bisset C. (2018). Appetite perceptions, gastrointestinal symptoms, ghrelin, peptide YY and state anxiety are disturbed in adolescent females with anorexia nervosa and only partially restored with short-term refeeding. Nutrients.

[68] Geliebter A., Melton P.M., McCray R.S., Gallagher D.R., Gage D., Hashim S.A. (1992). Gastric capacity, gastric emptying, and test-meal intake in normal and bulimic women. Am. J. Clin. Nutr..

[69] Chen J., Brady P. (2019). Gastroesophageal reflux disease: Pathophysiology, diagnosis, and treatment. Gastroenterol. Nurs..

[70] Herregods T.V., Bredenoord A.J., Smout A.J. (2015). Pathophysiology of gastroesophageal reflux disease: New understanding in a new era. Neurogastroenterol. Motil..

[71] de Bortoli N., Tolone S., Frazzoni M., Martinucci I., Sgherri G., Albano E., Ceccarelli L., Stasi C., Bellini M., Savarino V. (2018). Gastroesophageal reflux disease, functional dyspepsia and irritable bowel syndrome: Common overlapping gastrointestinal disorders. Ann. Gastroenterol..

[72] Barbera R., Feinle C., Read N.W. (1995). Abnormal sensitivity to duodenal lipid infusion in patients with functional dyspepsia. Eur. J. Gastroenterol. Hepatol..

[73] Feinle-Bisset C., Azpiroz F. (2013). Dietary and lifestyle factors in functional dyspepsia. Nat. Rev. Gastroenterol. Hepatol..

[74] Pilichiewicz A.N., Horowitz M., Holtmann G.J., Talley N.J., Feinle-Bisset C. (2009). Relationship between symptoms and dietary patterns in patients with functional dyspepsia. Clin. Gastroenterol. Hepatol..

[75] Bradette M., Pare P., Douville P., Morin A. (1991). Visceral perception in health and functional dyspepsia. Crossover study of gastric distension with placebo and domperidone. Dig. Dis. Sci..

[76] Mearin F., Cucala M., Azpiroz F., Malagelada J.R. (1991). The origin of symptoms on the brain-gut axis in functional dyspepsia. Gastroenterology.

[77] Caldarella M.P., Azpiroz F., Malagelada J.R. (2003). Antro-fundic dysfunctions in functional dyspepsia. Gastroenterology.

[78] Feinle-Bisset C., Meier B., Fried M., Beglinger C. (2003). Role of cognitive factors in symptom induction following high and low fat meals in patients with functional dyspepsia. Gut.

[79] Pilichiewicz A.N., Feltrin K.L., Horowitz M., Holtmann G., Wishart J.M., Jones K.L., Talley N.J., Feinle-Bisset C. (2008). Functional dyspepsia is associated with a greater symptomatic response to fat but not carbohydrate, increased fasting and postprandial CCK, and diminished PYY. Am. J. Gastroenterol..

[80] Houghton L.A., Mangall Y.F., Dwivedi A., Read N.W. (1993). Sensitivity to nutrients in patients with non-ulcer dyspepsia. Eur. J. Gastroenterol. Hepatol..

[81] Barbera R., Feinle C., Read N.W. (1995). Nutrient-specific modulation of gastric mechanosensitivity in patients with functional dyspepsia. Dig. Dis. Sci..

[82] Feinle C., Meier O., Otto B., D’Amato M., Fried M. (2001). Role of duodenal lipid and cholecystokinin A receptors in the pathophysiology of functional dyspepsia. Gut.

[83] Chua A.S., Dinan T.G., Rovati L.C., Keeling P.W. (1994). Cholecystokinin hyperresponsiveness in dysmotility-type nonulcer dyspepsia. Ann. N. Y. Acad. Sci..

[84] Azpiroz F., Bouin M., Camilleri M., Mayer E.A., Poitras P., Serra J., Spiller R.C. (2007). Mechanisms of hypersensitivity in IBS and functional disorders. Neurogastroenterol. Motil..

[85] Chey W.D., Kurlander J., Eswaran S. (2015). Irritable bowel syndrome: A clinical review. JAMA.

[86] Ukleja A. (2010). Altered GI motility in critically Ill patients: Current understanding of pathophysiology, clinical impact, and diagnostic approach. Nutr. Clin. Pract..

[87] Giezenaar C., Trahair L.G., Rigda R., Hutchison A.T., Feinle-Bisset C., Luscombe-Marsh N.D., Hausken T., Jones K.L., Horowitz M., Chapman I. (2015). Lesser suppression of energy intake by orally ingested whey protein in healthy older men compared with young controls. Am. J. Physiol. Regul. Integr. Comp. Physiol..

[88] Giezenaar C., van der Burgh Y., Lange K., Hatzinikolas S., Hausken T., Jones K.L., Horowitz M., Chapman I., Soenen S. (2018). Effects of substitution, and adding of carbohydrate and fat to whey-protein on energy intake, appetite, gastric emptying, glucose, insulin, ghrelin, CCK and GLP-1 in healthy older men-a randomized controlled trial. Nutrients.

[89] MacIntosh C.G., Morley J.E., Wishart J., Morris H., Jansen J.B., Horowitz M., Chapman I.M. (2001). Effect of exogenous cholecystokinin (CCK)-8 on food intake and plasma CCK, leptin, and insulin concentrations in older and young adults: Evidence for increased CCK activity as a cause of the anorexia of aging. J. Clin. Endocrinol. Metab..

[90] Di Francesco V., Zamboni M., Dioli A., Zoico E., Mazzali G., Omizzolo F., Bissoli L., Solerte S.B., Benini L., Bosello O. (2005). Delayed postprandial gastric emptying and impaired gallbladder contraction together with elevated cholecystokinin and peptide YY serum levels sustain satiety and inhibit hunger in healthy elderly persons. J. Gerontol. A Biol. Sci. Med. Sci..

[91] Horowitz M., Maddern G.J., Chatterton B.E., Collins P.J., Harding P.E., Shearman D.J. (1984). Changes in gastric emptying rates with age. Clin. Sci..

[92] Wegener M., Borsch G., Schaffstein J., Luth I., Rickels R., Ricken D. (1988). Effect of ageing on the gastro-intestinal transit of a lactulose-supplemented mixed solid-liquid meal in humans. Digestion.

[93] Clarkston W.K., Pantano M.M., Morley J.E., Horowitz M., Littlefield J.M., Burton F.R. (1997). Evidence for the anorexia of aging: Gastrointestinal transit and hunger in healthy elderly vs. young adults. Am. J. Physiol..

[94] Rayner C.K., MacIntosh C.G., Chapman I.M., Morley J.E., Horowitz M. (2000). Effects of age on proximal gastric motor and sensory function. Scand. J. Gastroenterol..

[95] Soenen S., Giezenaar C., Hutchison A.T., Horowitz M., Chapman I., Luscombe-Marsh N.D. (2014). Effects of intraduodenal protein on appetite, energy intake, and antropyloroduodenal motility in healthy older compared with young men in a randomized trial. Am. J. Clin. Nutr..

[96] Cook C.G., Andrews J.M., Jones K.L., Wittert G.A., Chapman I.M., Morley J.E., Horowitz M. (1997). Effects of small intestinal nutrient infusion on appetite and pyloric motility are modified by age. Am. J. Physiol..

[97] MacIntosh C.G., Andrews J.M., Jones K.L., Wishart J.M., Morris H.A., Jansen J.B., Morley J.E., Horowitz M., Chapman I.M. (1999). Effects of age on concentrations of plasma cholecystokinin, glucagon-like peptide 1, and peptide YY and their relation to appetite and pyloric motility. Am. J. Clin. Nutr..

[98] Sturm K., MacIntosh C.G., Parker B.A., Wishart J., Horowitz M., Chapman I.M. (2003). Appetite, food intake, and plasma concentrations of cholecystokinin, ghrelin, and other gastrointestinal hormones in undernourished older women and well-nourished young and older women. J. Clin. Endocrinol. Metab..

[99] Giezenaar C., Hutchison A.T., Luscombe-Marsh N.D., Chapman I., Horowitz M., Soenen S. (2017). Effect of age on blood glucose and plasma insulin, glucagon, ghrelin, CCK, GIP, and GLP-1 responses to whey protein ingestion. Nutrients.

[100] Rigamonti A.E., Pincelli A.I., Corra B., Viarengo R., Bonomo S.M., Galimberti D., Scacchi M., Scarpini E., Cavagnini F., Muller E.E. (2002). Plasma ghrelin concentrations in elderly subjects: Comparison with anorexic and obese patients. J. Endocrinol..

[101] Di Francesco V., Fantin F., Residori L., Bissoli L., Micciolo R., Zivelonghi A., Zoico E., Omizzolo F., Bosello O., Zamboni M. (2008). Effect of age on the dynamics of acylated ghrelin in fasting conditions and in response to a meal. J. Am. Geriatr. Soc..

[102] Di Francesco V., Zamboni M., Zoico E., Mazzali G., Dioli A., Omizzolo F., Bissoli L., Fantin F., Rizzotti P., Solerte S.B. (2006). Unbalanced serum leptin and ghrelin dynamics prolong postprandial satiety and inhibit hunger in healthy elderly: Another reason for the “anorexia of aging”. Am. J. Clin. Nutr..

[103] Wisen O., Johansson C. (1992). Gastrointestinal function in obesity: Motility, secretion, and absorption following a liquid test meal. Metabolism.

[104] Castiglione K.E., Read N.W., French S.J. (2002). Adaptation to high-fat diet accelerates emptying of fat but not carbohydrate test meals in humans. Am. J. Physiol. Regul. Integr. Comp. Physiol..

[105] Park M.I., Camilleri M., O’Connor H., Oenning L., Burton D., Stephens D., Zinsmeister A.R. (2007). Effect of different macronutrients in excess on gastric sensory and motor functions and appetite in normal-weight, overweight, and obese humans. Am. J. Clin. Nutr..

[106] French S.J., Murray B., Rumsey R.D., Fadzlin R., Read N.W. (1995). Adaptation to high-fat diets: Effects on eating behaviour and plasma cholecystokinin. Br. J. Nutr..

[107] Boyd K.A., O’Donovan D.G., Doran S., Wishart J., Chapman I.M., Horowitz M., Feinle C. (2003). High-fat diet effects on gut motility, hormone, and appetite responses to duodenal lipid in healthy men. Am. J. Physiol. Gastrointest. Liver Physiol..

[108] Brennan I.M., Seimon R.V., Luscombe-Marsh N.D., Otto B., Horowitz M., Feinle-Bisset C. (2011). Effects of acute dietary restriction on gut motor, hormone and energy intake responses to duodenal fat in obese men. Int. J. Obes..

[109] Seimon R.V., Taylor P., Little T.J., Noakes M., Standfield S., Clifton P.M., Horowitz M., Feinle-Bisset C. (2014). Effects of acute and longer-term dietary restriction on upper gut motility, hormone, appetite, and energy-intake responses to duodenal lipid in lean and obese men. Am. J. Clin. Nutr..

[110] Weigle D.S., Breen P.A., Matthys C.C., Callahan H.S., Meeuws K.E., Burden V.R., Purnell J.Q. (2005). A high-protein diet induces sustained reductions in appetite, ad libitum caloric intake, and body weight despite compensatory changes in diurnal plasma leptin and ghrelin concentrations. Am. J. Clin. Nutr..

[111] Leidy H.J., Clifton P.M., Astrup A., Wycherley T.P., Westerterp-Plantenga M.S., Luscombe-Marsh N.D., Woods S.C., Mattes R.D. (2015). The role of protein in weight loss and maintenance. Am. J. Clin. Nutr..

[112] Hutchison A.T., Feinle-Bisset C., Fitzgerald P.C., Standfield S., Horowitz M., Clifton P.M., Luscombe-Marsh N.D. (2015). Comparative effects of intraduodenal whey protein hydrolysate on antropyloroduodenal motility, gut hormones, glycemia, appetite, and energy intake in lean and obese men. Am. J. Clin. Nutr..

[113] Edelbroek M., Sun W.M., Horowitz M., Dent J., Smout A., Akkermans L. (1994). Stereospecific effects of intraduodenal tryptophan on pyloric and duodenal motility in humans. Scand. J. Gastroenterol..

[114] Ullrich S.S., Fitzgerald P.C.E., Giesbertz P., Steinert R.E., Horowitz M., Feinle-Bisset C. (2018). Effects of intragastric administration of tryptophan on the blood glucose response to a nutrient drink and energy intake, in lean and obese men. Nutrients.

[115] Pepino M.Y., Finkbeiner S., Beauchamp G.K., Mennella J.A. (2010). Obese women have lower monosodium glutamate taste sensitivity and prefer higher concentrations than do normal-weight women. Obesity.

[116] Tack J., Talley N.J. (2013). Functional dyspepsia--symptoms, definitions and validity of the Rome III criteria. Nat. Rev. Gastroenterol. Hepatol..

[117] Barbara G., Feinle-Bisset C., Ghoshal U.C., Quigley E.M., Santos J., Vanner S., Vergnolle N., Zoetendal E.G. (2016). The intestinal microenvironment and functional gastrointestinal disorders. Gastroenterology.

[118] Bisschops R., Karamanolis G., Arts J., Caenepeel P., Verbeke K., Janssens J., Tack J. (2008). Relationship between symptoms and ingestion of a meal in functional dyspepsia. Gut.

[119] Filipovic B.F., Randjelovic T., Kovacevic N., Milinic N., Markovic O., Gajic M., Filipovic B.R. (2011). Laboratory parameters and nutritional status in patients with functional dyspepsia. Eur. J. Intern. Med..

[120] Carvalho R.V., Lorena S.L., Almeida J.R., Mesquita M.A. (2010). Food intolerance, diet composition, and eating patterns in functional dyspepsia patients. Dig. Dis. Sci..

[121] Cuperus P., Keeling P.W., Gibney M.J. (1996). Eating patterns in functional dyspepsia: A case control study. Eur. J. Clin. Nutr..

[122] Goktas Z., Koklu S., Dikmen D., Ozturk O., Yilmaz B., Asil M., Korkmaz H., Tuna Y., Kekilli M., Karamanoglu Aksoy E. (2016). Nutritional habits in functional dyspepsia and its subgroups: A comparative study. Scand. J. Gastroenterol..

[123] Gibson P.R., Shepherd S.J. (2012). Food choice as a key management strategy for functional gastrointestinal symptoms. Am. J. Gastroenterol..

[124] Mullan A., Kavanagh P., O’Mahony P., Joy T., Gleeson F., Gibney M.J. (1994). Food and nutrient intakes and eating patterns in functional and organic dyspepsia. Eur. J. Clin. Nutr..

[125] Kaess H., Kellermann M., Castro A. (1988). Food intolerance in duodenal ulcer patients, non ulcer dyspeptic patients and healthy subjects. A prospective study. Klin. Wochenschr..

[126] Elli L., Tomba C., Branchi F., Roncoroni L., Lombardo V., Bardella M.T., Ferretti F., Conte D., Valiante F., Fini L. (2016). Evidence for the presence of non-celiac gluten sensitivity in patients with functional gastrointestinal symptoms: Results from a multicenter randomized double-blind placebo-controlled gluten challenge. Nutrients.

[127] Santolaria S., Alcedo J., Cuartero B., Diez I., Abascal M., Garcia-Prats M.D., Marigil M., Vera J., Ferrer M., Montoro M. (2013). Spectrum of gluten-sensitive enteropathy in patients with dysmotility-like dyspepsia. Gastroenterol. Hepatol..

[128] Saito Y.A., Locke G.R., Weaver A.L., Zinsmeister A.R., Talley N.J. (2005). Diet and functional gastrointestinal disorders: A population-based case-control study. Am. J. Gastroenterol..

[129] De Castro J.M. (1993). Age-related changes in spontaneous food intake and hunger in humans. Appetite.

[130] Bauer J., Biolo G., Cederholm T., Cesari M., Cruz-Jentoft A.J., Morley J.E., Phillips S., Sieber C., Stehle P., Teta D. (2013). Evidence-based recommendations for optimal dietary protein intake in older people: A position paper from the PROT-AGE Study Group. J. Am. Med. Dir. Assoc..

[131] Ryan M., Salle A., Favreau A.M., Simard G., Dumas J.F., Malthiery Y., Berrut G., Ritz P. (2004). Oral supplements differing in fat and carbohydrate content: Effect on the appetite and food intake of undernourished elderly patients. Clin. Nutr..

[132] Kupfer R.M., Heppell M., Haggith J.W., Bateman D.N. (1985). Gastric emptying and small-bowel transit rate in the elderly. J. Am. Geriatr. Soc..

[133] Rolls B.J., Dimeo K.A., Shide D.J. (1995). Age-related impairments in the regulation of food intake. Am. J. Clin. Nutr..

[134] Roberts S.B., Fuss P., Heyman M.B., Evans W.J., Tsay R., Rasmussen H., Fiatarone M., Cortiella J., Dallal G.E., Young V.R. (1994). Control of food intake in older men. JAMA.

[135] Milan A.M., Cameron-Smith D. (2015). Digestion and postprandial metabolism in the elderly. Adv. Food Nutr. Res..

[136] Poppitt S.D., McCormack D., Buffenstein R. (1998). Short-term effects of macronutrient preloads on appetite and energy intake in lean women. Physiol. Behav..

[137] Tylner S., Cederholm T., Faxen-Irving G. (2016). Effects on weight, blood lipids, serum fatty acid profile and coagulation by an energy-dense formula to older care residents: A randomized controlled crossover trial. J. Am. Med. Dir. Assoc..

[138] Spiller R.C., Trotman I.F., Adrian T.E., Bloom S.R., Misiewicz J.J., Silk D.B. (1988). Further characterisation of the ’ileal brake’ reflex in man--effect of ileal infusion of partial digests of fat, protein, and starch on jejunal motility and release of neurotensin, enteroglucagon, and peptide YY. Gut.

